# M6ATMR: identifying N6-methyladenosine sites through RNA sequence similarity matrix reconstruction guided by Transformer

**DOI:** 10.7717/peerj.15899

**Published:** 2023-09-11

**Authors:** Shuang Xiang, Te Zhang, Minghao Wu

**Affiliations:** Changjiang Water Resources and Hydropower Development Group, Wuhan, Hubei, China

**Keywords:** RNA modification, N6-methyladenosine, Transformer, Similarity matrix, Graph

## Abstract

Numerous studies have focused on the classification of N6-methyladenosine (m6A) modification sites in RNA sequences, treating it as a multi-feature extraction task. In these studies, the incorporation of physicochemical properties of nucleotides has been applied to enhance recognition efficacy. However, the introduction of excessive supplementary information may introduce noise to the RNA sequence features, and the utilization of sequence similarity information remains underexplored. In this research, we present a novel method for RNA m6A modification site recognition called M6ATMR. Our approach relies solely on sequence information, leveraging Transformer to guide the reconstruction of the sequence similarity matrix, thereby enhancing feature representation. Initially, M6ATMR encodes RNA sequences using 3-mers to generate the sequence similarity matrix. Meanwhile, Transformer is applied to extract sequence structure graphs for each RNA sequence. Subsequently, to capture low-dimensional representations of similarity matrices and structure graphs, we introduce a graph self-correlation convolution block. These representations are then fused and reconstructed through the local-global fusion block. Notably, we adopt iteratively updated sequence structure graphs to continuously optimize the similarity matrix, thereby constraining the end-to-end feature extraction process. Finally, we employ the random forest (RF) algorithm for identifying m6A modification sites based on the reconstructed features. Experimental results demonstrate that M6ATMR achieves promising performance by solely utilizing RNA sequences for m6A modification site identification. Our proposed method can be considered an effective complement to existing RNA m6A modification site recognition approaches.

## Introduction

To date, approximately 160 chemical modifications have been discerned in RNA, substantially enriching the diversity of RNA function and genetic information (Rehman et al., 2021). Among these modifications, N6-methyladenosine (m6A) stands out as the most prevalent modification type in eukaryotes and the sole dynamic, reversible RNA modification, along with N1-methyladenosine (Wang & Yan, 2018). m6A plays pivotal roles in various cellular processes, including cell growth, mRNA selective splicing, stem cell differentiation, and circadian clock control (Fustin et al., 2013; Geula et al., 2015; Wang & Wang, 2020; Wang et al., 2014; Wang et al., 2018). Furthermore, m6A exhibits close associations with the pathogenesis of diverse diseases, such as prostate cancer, acute myeloid leukemia, and thyroid tumors (Rehman et al., 2021). Given the significance of m6A, there exists an imperative to identify potential m6A modification sites.

High-throughput sequencing techniques have been extensively utilized for the identification of m6A modification sites, including m6A sequencing (Dominissini et al., 2012), crosslinking immunoprecipitation (Ke et al., 2015), and Methylated RNA Immunoprecipitation (Meyer et al., 2012). These methodologies have significantly contributed to our understanding of m6A modification on RNA. However, due to the dynamic and tissue-specific nature of m6A modification sites, limited experimental approaches may not be sufficiently flexible in identifying potential modification sites (Wang & Yan, 2018). Furthermore, the wet-lab experiments employed to identify m6A modification sites are often costly and time-consuming. In recent years, an increasing number of studies have recognized the notable advantages of computational methods for identifying RNA m6A modification sites. These computational approaches offer high generalization, rapid processing times, and lower costs, rendering them an attractive and viable alternative.

The computational identification of RNA m6A modification sites can be broadly categorized into two groups: machine learning-based methods and deep learning-based methods. Both approaches share a common core, which is the feature extraction task, aimed at capturing better representations of RNA sequences for improved recognition performance. Machine learning-based methods typically involve two main stages: feature engineering and downstream classification. In feature engineering, various coding approaches have been applied to represent RNA sequences, including k-mer, one-hot coding, accumulated nucleotide frequency (Chen et al., 2015), composition of k-space nucleic acid pairs (Zhang et al., 2020), dinucleotide composition (Di Giallonardo et al., 2017), and enhanced nucleic acid composition (Huang et al., 2018). The iRNA toolkits (Chen et al., 2018; Qiu et al., 2017; Yang et al., 2018) are noteworthy examples that utilize these encoding methods. Moreover, the iRNA toolkits were the pioneers in incorporating the physicochemical properties of nucleotides for recognizing various types of RNA modification sites. In the downstream classification task, different classifiers are usually employed to recognize the extracted features, including random forest (RF) (Breiman, 2001). The iRNA toolkits, AthMethPre (Xiang et al., 2016), M6ATH (Chen et al., 2016), and RAM-NPPS (Xing et al., 2017) prefer Support Vector Machine (SVM), while other methods like SRAMP (Zhou et al., 2016) and M6AMRFS (Qiang et al., 2018) explore ensemble methods with multiple classifiers for downstream tasks. On the other hand, deep learning-based methods view feature extraction and classification as continuous processes, allowing them to learn more semantic information from the data. Researchers are increasingly turning to deep learning strategies for RNA m6A modification site recognition tasks. For example, iN6-Methyl (Nazari et al., 2019) and m6AGE (Wang et al., 2021) use convolutional neural networks to extract sequence features.

These studies have made significant progress in identifying RNA m6A modification sites. However, they also have limitations. For instance, incorporating additional information, like the physicochemical properties of nucleotides, alongside RNA sequences may introduce potential information interference. Moreover, these methods have mainly focused on learning features from sequential nucleotide distributions, potentially overlooking associations of nucleotides through self-correlations in RNA sequences. To address these issues, we propose a novel approach in this article, named m6ATMR, for RNA m6A modification site recognition. m6ATMR utilizes Transformer (Vaswani et al., 2017) to guide the reconstruction of the nucleotide similarity matrix, thereby enhancing feature representations of RNA sequences in a sequence-dependent manner. Specifically, RNA sequences are first encoded using the 3-mer method, generating the initial similarity matrix for each sequence. Then, Transformer is applied to further obtain the sequence structure graphs of RNA sequences. To optimize the sequence structure graphs, we calculate the Manhattan distance and perform threshold screening on the vector representation from Transformer. Next, we design a graph self-correlation convolution block to obtain low-dimensional representations of both the similarity matrix and the structure graph. In addition, we dynamically combine the low-dimensional representations obtained from the initial 3-mer representations of RNA sequences, considering both local and global perspectives, to create the final recombined features. To explore potential nucleotide associations in RNA sequences, we use iteratively updated sequence structure graphs to continuously optimize the similarity matrices, further enhancing the end-to-end feature extraction process. Finally, we employ the random forest (RF) algorithm to classify and recognize RNA sequences based on the learned features. By following this approach, m6ATMR aims to overcome the limitations of previous methods and improve the accuracy of RNA m6A modification site identification.

The main contributions of this article are as follows: First, we propose a sequence-based approach for identifying RNA m6A modification sites without introducing potentially misleading additional information. Second, the similarity matrices of RNA sequences are computed to provide more effective information that can be learned for sequences, and on this basis, the Transformer is used to reconstruct the similarity matrices and further optimize the sequence representations. Third, we propose a graph self-correlation convolution to learn a low-dimensional representation of the sequence without introducing prior information about the nodes. A series of experiments demonstrate the effectiveness of the representation strategies of M6ATMR. For M6ATMR, it is worth noting that relying solely on RNA sequences for m6A modification site identification can reduce the need for additional prior information, while still ensuring high identification accuracy. In addition, the sequence representations learned by our model perform consistently well across different classifiers, indicating that our method is not dependent on the choice of a specific classifier. In conclusion, our experimental results demonstrate that M6ATMR achieves excellent performance in identifying m6A modification sites using only RNA sequences. This highlights its effectiveness as a complementary method for RNA m6A modification site recognition.

## Materials and Methods

### Problem description and datasets

One of the focuses of RNA modification research is site recognition. For this task, the computational methods are usually to convert the problem into a binary classification problem, which takes the RNA sequence information as the initial input to the classification model and gets the probability value of modification sites. The method in our article follows this paradigm. For a given sequence ${\rm X} = \left\{ {{x_1},{x_2} \ldots {x_n}} \right\}$, we summarize the model objective as ${\rm Y} = {f_\sigma }\left( X \right) \in \left\{ {0,1} \right\}$, where ${f_\sigma }\left( \cdot \right)$ is the optimal mapping relationship, and ${\rm Y}$ is the prediction label. If the prediction result is a positive sample, ${\rm Y} = 1$, otherwise ${\rm Y} = 0$. It is worth noting that, as in most studies, the inclusion of m6A modification sites is used as the classification criteria for positive and negative samples. That is, for a given sequence ${\rm X}$, if its central position is the modified site, sequence ${\rm X}$ is regarded as a positive sample. Negative samples do not contain modification sites. To this end, we select the RNA m6A modification site dataset of *Arabidopsis thaliana* (A101 dataset) (Wan et al., 2015) for study. The dataset contains 2,100 samples, in which the ratio of positive and negative samples is 1:1.

### Model description

**Figure 1:**
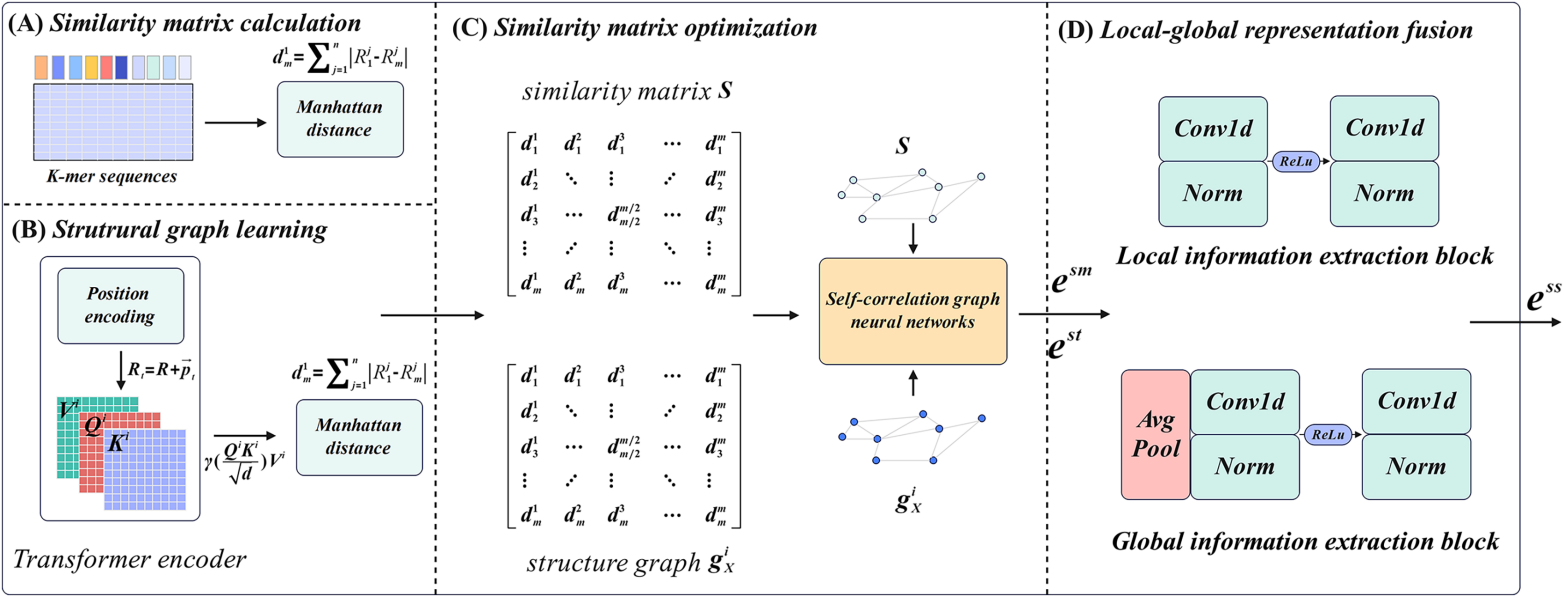
The framework of M6ATMR. (A) The details of similarity matrix calculation process. (B) The details of structural graph learning process with the Transformer encoder. (C) The similarity matrix optimization process with the self-correlation graph neural networks. (D) The structure of the local-global representation fusion block.

M6ATMR implements the identification task of m6A modification sites based on several procedures. First, the RNA sequences were processed into readable coding representation based on k-mer algorithm, and the similarity between RNA sequences was measured by Manhattan distance to further construct the similarity matrix. After that, M6ATMR learned the structural information of RNA sequences through the Transformer encoder, and converts this structural information into the structural graph by Manhattan distance. It then inputted the similarity matrix and structure graph into the self-correlation graph neural network to iteratively update the similarity matrix and obtain the low-dimensional representations of each RNA sequence. These low-dimensional representations were passed through a local-global fusion block to generate the final fusion representation, which was fed into the RF for identification. For the convenience of description, section 2 describes M6ATMR in detail from four parts: similarity matrix calculation, structure graph learning, similarity matrix optimization, and local-global representation fusion. The framework of M6ATMR is shown in Fig. 1, and the details of each part are as follows.

### Similarity matrix calculation

For similarity matrix calculation, the key step is to transform RNA sequences into the readable representations using k-mer frequency statistics. Statistical k-mer frequency information can reveal the distribution law of seed sequences and is an important tool to study sequence similarity. In sequence ${\rm X}$, a substring of length $K$ refers to $K$ monometric units starting from any position in ${\rm X}$, which is called k-mer. In this article, considering that each of the three adjacent nucleotides in an mRNA molecule is organized into a group that represents the pattern of a particular amino acid in protein synthesis, therefore, to make the model biological interpretation, we set $K$ to 3. The algorithm requires that the starting position of the sequence set ${\Omega } = \left\{ {{X_1},{X_2}, \ldots ,{X_m}} \right\}$ is aligned. For k-mer sequences with fixed $K$ value, at the offset position $l\; \left( {0 \le l \le n - k} \right)$, we count the occurrence frequency of different substrings in k-mer sequence with length $K$ starting from the offset position $l$:

(1) $$R_{X} = \textit{Nom}\left(\oplus_{i = l}^{n - k}\;\textit{fre}\;\left(M_{i}\right)\right)$$

where $R_{X} \in {\mathbb{R}}^{n \times 1}$ denotes the k-mer representation of sequence $X$, and $fre\left( \cdot \right)$ represents the frequency of the k-mer substring ${M_i}$, and $\oplus$ represents the operation of concatenating all substring frequencies. $Nom\left( \cdot \right)$ represents a normalized operation.

After representing all RNA sequences using statistical k-mer frequency, we attempt to calculate the similarity between each sequence and construct an initial similarity matrix of RNA sequences. We construct matrices between k-mer representations for the following reasons: Firstly, earlier studies on the prediction of biomedical entity associations (Shao et al., 2020) have shown that similarity matrices can reflect the potential association between different entities, which provides strong support for the establishment of the association between different sequences of nucleotides. Secondly, constructing a graph data structure based on the similarity matrix allows us to capture the relationships between multiple sequences, which creates conditions for further sequence extraction. Therefore, it is a reasonable choice to transform the sequence representing the problem into the optimization task of the similarity matrix. In this article, we choose to employ the Manhattan distance to further measure the degree of similarity between nucleotides on different sequences:

(2) $$S = \left( \matrix{{d_1^m} & \cdots & {d_1^m} \\ \vdots & \ddots & \vdots \\ d_{m}^{1} & \cdots & {d_m^m} } \right)$$

where, in the similar matrix $\text{S} \in {\mathbb{R}}^{m \times m}$, each element represents the Manhattan distance between k-mer sequences of corresponding positions. Taking $d_1^m$ as an example, its value is the Manhattan distance between sequence representation ${R_1}$ and ${R_m}$:

(3) $$d_m^1 = \mathop \sum \nolimits_{j = 1}^n \left| {R_1^j - R_m^j} \right|$$

where, the $R_1^j$, $R_m^j$ represent the $jth$ k-mer value in sequence state ${R_1}$ and ${R_m}$ respectively, and $\left| \cdot \right|$ denotes the operation of taking the absolute value.

### Structure graph learning

When obtaining the initial similarity matrices of the RNA sequences, we use a Transformer encoder to capture the structural information and learn the structural graph of RNA sequences. To this end, we apply the encoder part of the Transformer to further process the k-mer sequence. For a given Transformer encoder, there are two basic components: the position encoding block, and the self-attention mechanism. In addition, in order to further explore the structural relationship between RNA sequences, we also calculate the Manhattan distance between vector representations of the output of the Transformer encoding block, and strictly constrain the value of the structure matrix within the set of $\left\{ {0,1} \right\}$. The details are as follows.

Position encoding of Transformer is a functional encoder, that is, position vectors are calculated for each element in the sequence:

(4) $$\matrix{ {{{\overrightarrow {{p_t}} }^{\left( i \right)}} = f{{\left( t \right)}^{\left( i \right)}}:= \bigg\{ {\matrix{ {\; \sin \left( {{w_i}\cdot t} \right)\; \; i = 2k\; \; \; \; } \cr {\cos \left( {{w_i}\cdot t} \right)\; \; i = 2k + 1} } } } }$$

where ${w_i}$ denotes the frequency, which is calculated as follows:

(5) $${w_i} = \displaystyle{1 \over {{{10000}^{2i/d}}}}$$

where $d$ is the output dimension of the neural network. It is worth noting that the length of this position vector is equal to the length of k-mer representations. Thus, the RNA sequences are represented by k-mer and position encoding:

(6) $${R_t} = R + \overrightarrow {{p_t}}$$

where, $\overrightarrow {{p_t}}$ denotes the position vectors of all elements in k-mer representations. After calculating the position vectors, the encoder further optimizes the representation ${R_t}$ through the self-attention mechanism:

(7) $$\left\{ {\matrix{ {{Q^i} = {w^q}{X^i}\; \; \; \; \; \; \; \; \; \; \; \; \; \; } \cr {{K^i} = {w^k}{X^i}\; \; \; \; \; \; \; \; \; \; \; \; \; \; } \cr {{V^i} = {w^v}{X^i}\; \; \; \; \; \; \; \; \; \; \; \; \; \; } \cr {g_X^i = \gamma \left( {\displaystyle{{{Q^i}{{({K^i})}^T}} \over {\sqrt d }}} \right){V^i}\; \; \; \; \; \; \; \; \; } \cr } } \right.$$

where, ${Q^i}$, ${K^i}$ and ${V^i}$ are the query matrix, key matrix and value matrix respectively. ${\rm \gamma }$ denotes the $softmax$ activation function. Through the self-attention mechanism, the Encoder can reveal the potential associations within the sequence and further excavate the structural associations of nucleotides.

In this regard, we believe that it is a valid way to describe the structural association of RNA sequences through internal relationships captured by the self-attention mechanism. To this end, we also measure this structural associations by the same method in section 2.3 and construct the structure graph ${G_{st}}$ for sequences. Since the model samples the same data and distance formulas during the calculation process, the structure graph has a potential correlation with the similarity matrix, which indicates that it is reasonable to optimize the similarity matrix further through ${G_{st}}$. In addition, we ensure that the values of elements in ${G_{st}}$ are strictly constrained in the set of $\left\{ {0,1} \right\}$ by threshold filtering, as shown below:

(8) $$G_{st}^{\left( {i,j} \right)} = \left\{ {\matrix{ {1\; \; \; \; if\; G\left( {i,j} \right) \gt 0.5} \cr \!\!\!\!\!\!\!{0\; \; \; \; otherwise\; \; \; \; } \cr } } \right.$$

For each element $G\left( {i,j} \right)$ in $G_{st}^{\left( {i,j} \right)}$, updating the value to 0 if $G\left( {i,j} \right)$ is less than 0.5, otherwise updating the value to 1.

### Similarity matrix optimization

To optimize the sequence similarity matrix and update the sequence structure graph, we design a self-correlation graph neural network that does not depend on prior node representations. In traditional graph neural networks, the embedded learning process relies on the existing representations of the nodes or edges in the graph. These prior representations serve as the starting point for the learning algorithm to update and refine the embeddings based on the graph structure. For instance, in some studies related to drug-drug association prediction, the SMILES of drugs are often applied as the prior information to serve as the initial input to the graph neural network. However, for similar matrix and structure graphs of sequences, the initial information of nodes is difficult to be obtained. In addition, both the similarity matrix and the structure graph describe the self-correlation property of the sequence, which makes the introduction of additional prior information may lead to misleading information. Therefore, inspired by the self-attention mechanism, we generate the learnable initial node representations based on the input matrix information. The initial representations are constantly updated and optimized during the process of graph convolution and participate in the optimization of the final embeddings. Taking the processing of similarity matrix as an example, we describe in detail the learning process of representation of similarity graph.

For a given similarity matrix ${S} \in {\mathbb{R}}^{m \times m}$, we define the node initial representation matrix as $Er \in{\mathbb{R}}^{m \times m}$. Each value in the matrix is determined and iteratively optimized by the network. $S$ and $Er$ are input into the three-layer self-correlation graph neural networks to get the embeddings related to the similarity matrix $S$:

(9) $$\matrix{ {\left\{ {\matrix{ {R_s^{\left( {i + 1} \right)} = R_s^{\left( i \right)} + {\alpha ^{\left( {i + 1} \right)}}Gcov{{\left( {Er,S} \right)}^{\left( {i + 1} \right)}}} \cr {{\alpha ^{\left( {i + 1} \right)}} = \displaystyle{1 \over {I + 1}}\left( {i + 1} \right)\; \; \; \; \; \; \; \; \; \; \; \; \; \; \; } \cr } } \right.}}$$

where, $\alpha$ is the scaling superparameter to prevent the elements in the similarity matrix representation from becoming infinitesimal during graph convolution, and $I = 3$ denotes the number of convolution layers. $Gcov\left( \cdot \right)$ represents the convolution process. The hidden layer representation of layer $\left( {i + 1} \right)$ and the representation of layer $\left( i \right)$ satisfies the following equation:

(10) $${H^{\left( {l + 1} \right)}} = \sigma ({S^{ - \displaystyle{1 \over 2}}}\left( {diag\left( {D{e_s}} \right) - \displaystyle{1 \over 2}\left( {S + {S^T}} \right)){S^{ - \displaystyle{1 \over 2}}}{H^{\left( l \right)}}{W^{\left( l \right)}}} \right)$$

where $D{e_s}$ denotes the degree matrix of the similar matrix $S$, and $diag\left( \cdot \right)$ denotes the diagonalization operation. $W$ is the learnable weight. The representation of the hidden layer is initialized to $Er$, that is, ${H^{\left( 0 \right)}} = Er$. Similarly, sequence structure graphs are fed into the self-correlation neural networks in the same way. The network further employs learnable initial representations to mine the self-correlation of sequences, which also ensures consistency between representations learned from similarity matrices and that learned from structure graphs.

In addition, another task of the networks is to get better RNA representations by optimizing the similarity matrix. Hence, we apply the reconstructed similarity matrix and sequence structure graph to calculate the loss of the networks:

(11) $${\rm {\cal L}}\left( {\hat S,S,\widehat {{G_{st}}},{G_{st}}} \right) = BCELoss(\hat S,{\rm sigm}\left( {{\rm S*}{S^T}} \right) + BCELoss(\widehat {{G_{st}}},{\rm sigm}\left( {{G_{st}}{\rm *}{G_{st}}^T} \right)$$

where $BCELoss\left( \cdot \right)$ denotes the binary cross-entropy loss. In the optimization process, we employ the difference between the reconstructed element values and the original element values to measure the performance of the representations.

### Local-global representation fusion

In order to obtain comprehensive knowledge of RNA sequences, we propose a local-global strategy for fusing learned embedded representations. We process two kinds of representations of the same sequence respectively from the local and global perspectives of embedding representations, and further determine the weight relationship between embedding representations from similarity matrices and embedding representations from sequence structure graphs. Specifically, we design a local information extraction block and a global information extraction block respectively to calculate the weights of the two types of embedded representations.

For a given similarity matrix embedding representation ${e^{sm}}$ and structure graph embedding representation ${e^{st}}$, we first treat them as residue sequences and weight them to obtain an overall representation ${e^a}$:

(12) $${e^a} = {e^{sm}} + {e^{st}}$$

We then enter ${e^a}$ into the local and global information extraction blocks. For the local and global information extraction block, the extraction process is described as follows:

(13) $$\left\{ {\matrix{
{e_{lo}^a = f\left( {\vartheta \left( {{f^\prime }\left( {{e^a}} \right)} \right)} \right)}  \cr 
{e_{gl}^a = f\left( {\vartheta \left( {{f^\prime }\left( {\delta \left( {{e^a}} \right)} \right)} \right)} \right)}  \cr 

} } \right.$$

where $e_{lo}^a$ and $e_{gl}^a$ denote the output of the local extraction block and that of the global extraction block respectively. $f$ and ${f}^{\prime}$ represent a one-dimensional convolution layer containing normalized functions respectively, and $\vartheta$ represents the $ReLu$ function. For the global information extraction block, we add the global average pooling layer $\delta$ on the basis of the local information extraction block. After that, we further calculate the weight difference ${w_f}$ between the two representations:

(14) $${w_f} = sigm\left( {e_{lo}^a + e_{gl}^a} \right)$$

We utilize this weight difference to further integrate the two types of embedded representations to obtain the similar-structural representation ${e^{ss}}$:

(15) $${e^{ss}} = {w_f}*{e^{sm}} + \left( {1 - {w_f}} \right)*{e^{st}}$$

In addition, we consider the indispensable role of the k-mer representations of the sequences for the recognition of m6A modification sites, and further integrate these representations with the similar-structural representations to obtain the final embedding representations ${e^{fi}}$:

(16) $${e^{fi}} = {w_{{f}^{\prime}}}*{e^{ss}} + \left( {1 - {w_{{f}^{\prime}}}} \right)*{e^{km}}$$

where, ${e^{km}}$ denotes the K-mer representations of the sequences, and ${w_{{f}^{\prime}}}$ is the weight difference between ${e^{km}}$ and ${e^{ss}}$.

### Experiments setting

For a binary classification problem, its prediction states can be divided into the four categories: true positive (TP), false positive (FP), true negative (TN), false negative (FN). Thus, we select some predictive indicators to evaluate the prediction effect, and the calculation processes of these indicators are as below. Moreover, we obtain the area under the precision-recall curve (AUPR) and area under the receiver-operating characteristic curve (AUC) for evaluating our model.

(17) $$Accuracy\; \left( {Acc} \right) = \displaystyle{{TP + TN} \over {TP + TN + FP + FN}}$$

(18) $${ {F1 = \displaystyle{{prec \times Sn} \over {prec + Sn}}} }$$

(19) $$Precision\; \left( {Prec} \right) = \displaystyle{{TP} \over {TP + FN}}$$

(20) $$Sensitivity\; \left( {Sn} \right) = \displaystyle{{TP} \over {TP + FN}}$$

(21) $$Specificity\; \left( {Sp} \right) = \displaystyle{{TN} \over {TN + FP}}$$

(22) $$\matrix{ {Matthews\; Correlation\; Coefficient\; \left( {MCC} \right)} \cr { = \displaystyle{{TP \times TN - FP \times FN} \over {\sqrt {\left( {TP + FP} \right)\left( {TP + FN} \right)\left( {TN + FP} \right)\left( {TN + FN} \right)} }}} }$$

In our study, we set the learning rate to 0.0001 and set the number of layers as three in the self-correlation graph neural network. Considering the time and complexity of training, we reduced the number of heads from eight to six in the Transformer encoder. We set the embedding size of the encoder to 32, the hidden dimension of the feed-forward layer to 128, and the number of encoder blocks to six.

## Results

### Performance on A101 datasets

**Figure 2:**
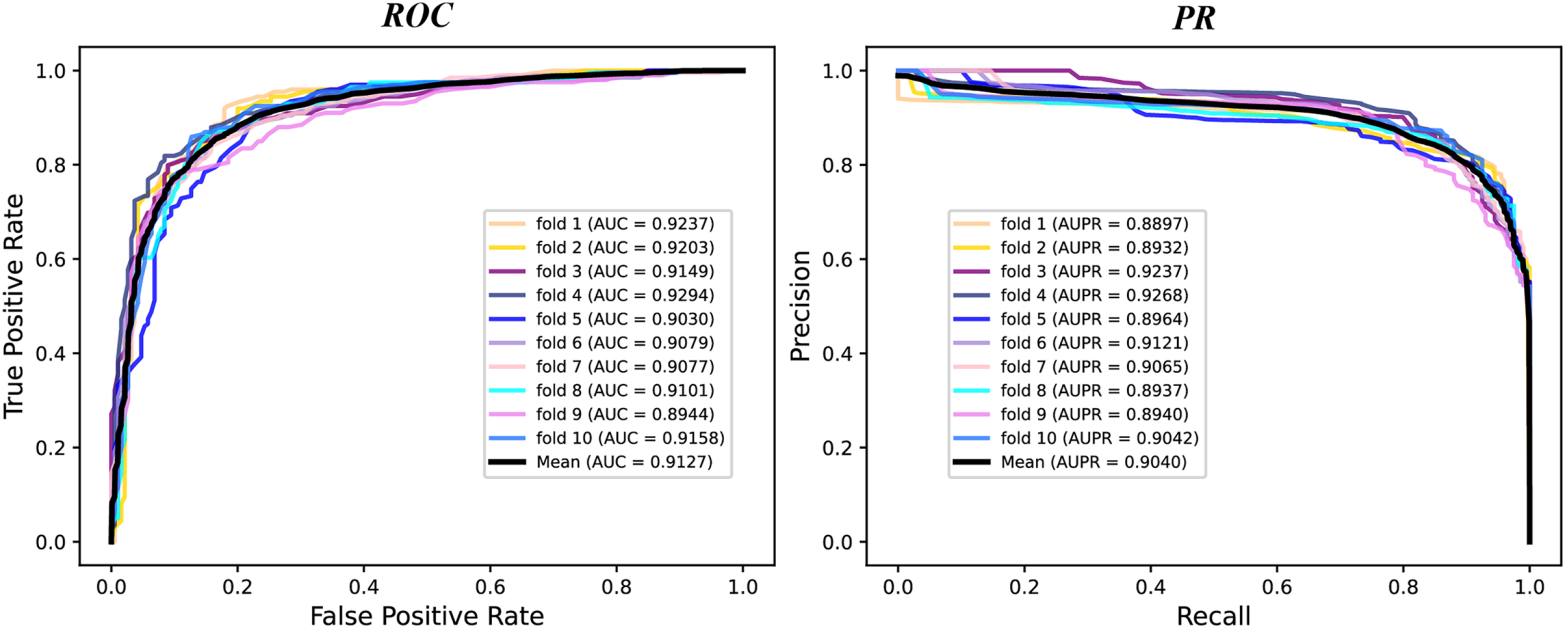
The ROC curves and PR curves of M6ATMR on A101 dataset under 10-fold cross-validation.

We conduct a rigorous 10-fold cross validation to evaluate the performance of our proposed model on the A101 dataset. The dataset is systematically partitioned into ten subsets of equal size, ensuring non-overlapping test sets in each fold. For each fold, we utilize nine subsets for training and one for testing. During evaluation, we consider six essential indicators: AUPR, AUC, Acc, F1 score, Prec, and Sen. The results of the 10-fold cross validation are presented through PR curves and ROC curves in Fig. 2 and summarized in Table 1. Across the 10 folds, the AUC curve demonstrates remarkable stability, with the maximum AUC reaching 93.87% and the minimum at 89.79%. Similarly, the PR curve displays consistent performance, with the highest AUPR at 93.17% and the lowest at 87.66%. Table 1 reveals outstanding performance in various evaluation metrics. The Acc achieves an impressive 84.43%, signifying a high correct identification rate for both TN and TP samples. Additionally, the MCC attains a value of 83.72%, reflecting the overall strength of our model. Furthermore, the values of other metrics, including F1 score, Prec, and Sen, surpass 80%, indicating the reliability of our model. These experimental findings substantiate the robustness and efficacy of our proposed model in identifying m6A modification sites.

**Table 1:** The value of some indicators in each fold.

Fold	MCC	Acc	Sn	Sp	Prec	F1	AUC	AUPR
0	0.7068	0.8535	0.8693	0.8368	0.8480	0.8586	0.9237	0.8897
1	0.7068	0.8535	0.8693	0.8368	0.8480	0.8586	0.9203	0.8932
2	0.6913	0.8458	0.8543	0.8368	0.8458	0.8500	0.9149	0.9237
3	0.7068	0.8535	0.8643	0.8421	0.8515	0.8579	0.9294	0.9268
4	0.6402	0.8201	0.8141	0.8263	0.8308	0.8223	0.9030	0.8964
5	0.6965	0.8483	0.8543	0.8421	0.8500	0.8521	0.9079	0.9121
6	0.6711	0.8355	0.8291	0.8421	0.8462	0.8376	0.9077	0.9065
7	0.7070	0.8535	0.8492	0.8579	0.8622	0.8557	0.9101	0.8937
8	0.6297	0.8149	0.8150	0.8148	0.8232	0.8191	0.8944	0.8940
9	0.7111	0.8557	0.8693	0.8413	0.8522	0.8607	0.9158	0.9042
Mean	0.6867	0.8434	0.8488	0.8377	0.8458	0.8473	0.9127	0.9040

### Performance comparison of Classifiers

**Figure 3:**
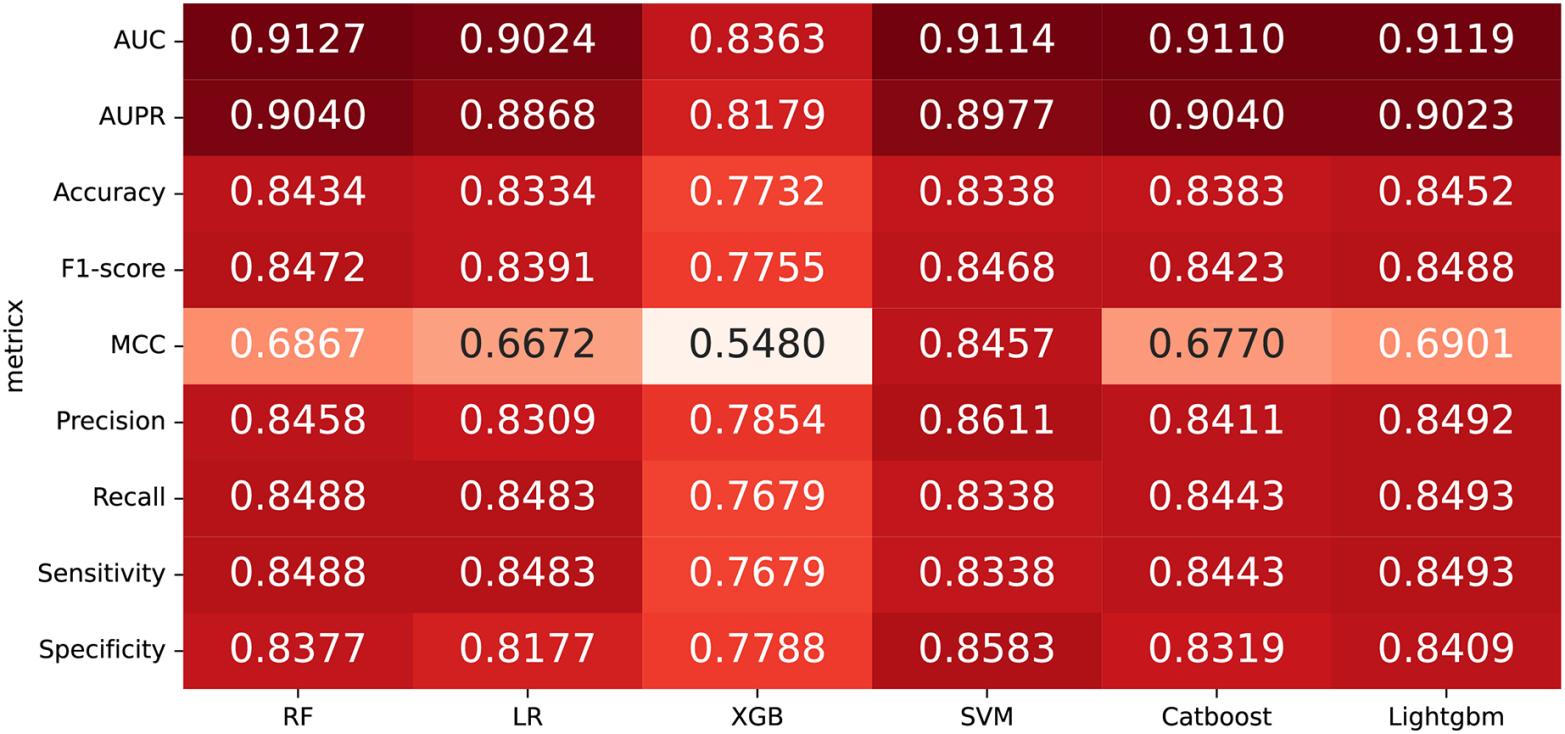
The comparison results of different classifiers.

**Figure 4:**
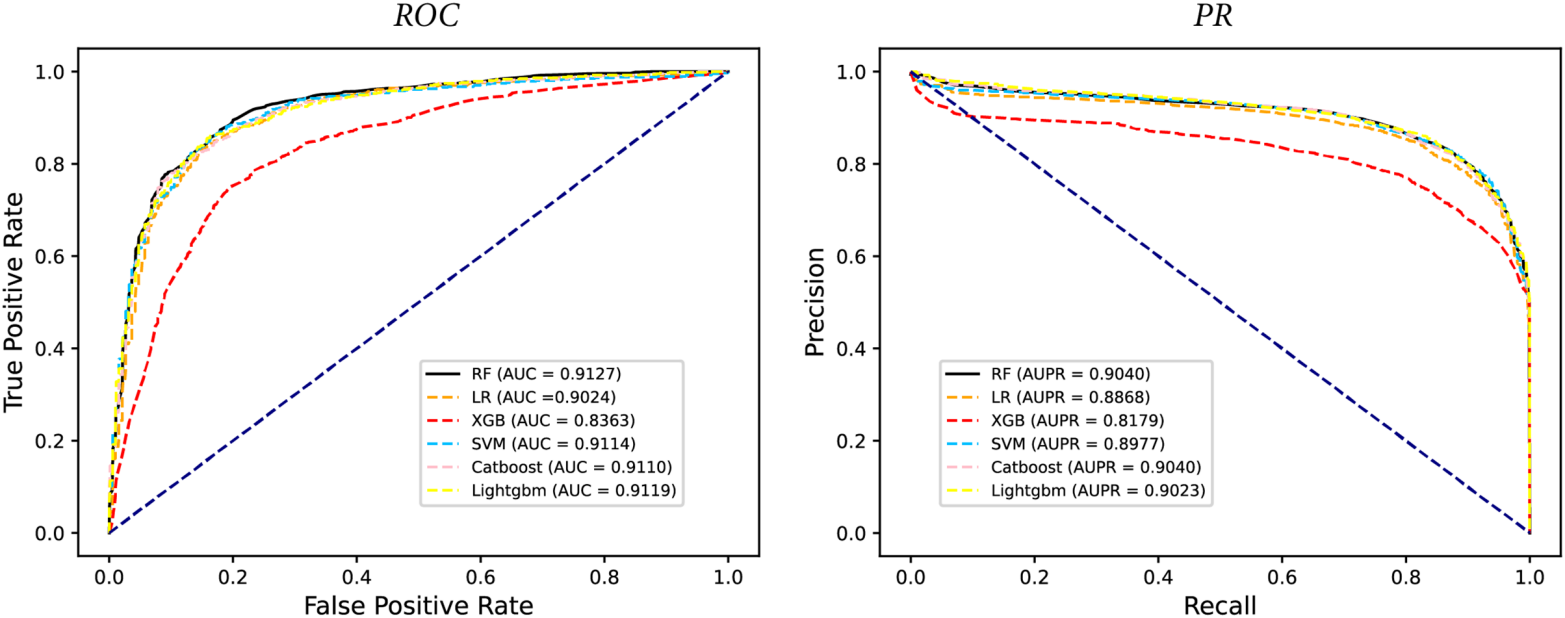
The PR curves of M6ATMR with different classifiers.

In some studies, the downstream classifiers have shown to significantly influence the classification performance of RNA sequence representations generated by models, potentially leading to model instability. To demonstrate the stability and efficacy of our proposed model, we conduct a comparison experiments using five additional classifiers: logistic regression, eXtreme Gradient Boosting (XGBoost), Light Gradient Boosting Machine (lightgbm), CatBoost, and support vector machines (SVM). Logistic regression employs maximum likelihood estimation to predict model parameters, yielding binary results by minimizing cross-entropy loss during data training. XGBoost, an integrated classification algorithm, employs multiple simple base learners to iteratively train input data, continually reducing the discrepancy between model and input values. In contrast, lightgbm stands out with its advantage of low memory usage and faster training speed. CatBoost is designed to extract the most information from given data and is particularly effective for small machine-learning datasets. SVM, as a binary classification model, aims to find an optimal hyperplane for sample segmentation. For evaluation, we utilize 10-fold cross validation on the A101 dataset, as mentioned in “Performance on A101 datasets”. The same set of indicators, AUPR, AUC, Acc, F1 score, Prec, and Sen are employed for assessment. The experimental results, presented in Fig. 3, Fig. 4, and Table 2, indicate that when RF is used as the downstream classifier, the model achieves the highest performance, with AUC at 91.27% and AUPR at 90.40%. While XGBoost exhibits relatively inferior performance compared to logistic regression and RF, the overall classification effect of all three classifiers remains relatively favorable. The difference in AUC values between XGBoost and RF is 7.84%. These findings support the notion that the RNA sequence representations learned by our model are not easily influenced by the choice of downstream classifiers, indicating the model’s stability and effectiveness in feature extraction and m6A modification site identification.

**Table 2:** The AUC value of some indicators in each fold based on different classifiers.

Fold	RF	LR	XGBoost	SVM	CatBoost	Lightgbm
Fold1	0.9237	0.9052	0.8454	0.9077	0.8951	0.9111
Fold2	0.9203	0.8932	0.8319	0.9085	0.9484	0.9305
Fold3	0.9149	0.9148	0.8125	0.8856	0.8928	0.8985
Fold4	0.9294	0.8806	0.8714	0.9270	0.9078	0.9080
Fold5	0.9030	0.9282	0.7987	0.9216	0.9089	0.8996
Fold6	0.90793	0.8855	0.8208	0.9033	0.9040	0.8752
Fold7	0.9077	0.9025	0.7991	0.9141	0.9284	0.9255
Fold8	0.9101	0.9022	0.8470	0.9108	0.8961	0.9412
Fold9	0.8944	0.9030	0.8821	0.9138	0.9195	0.9248
Fold10	0.9158	0.9087	0.8537	0.9290	0.9094	0.9052

### Features selection

**Figure 5:**
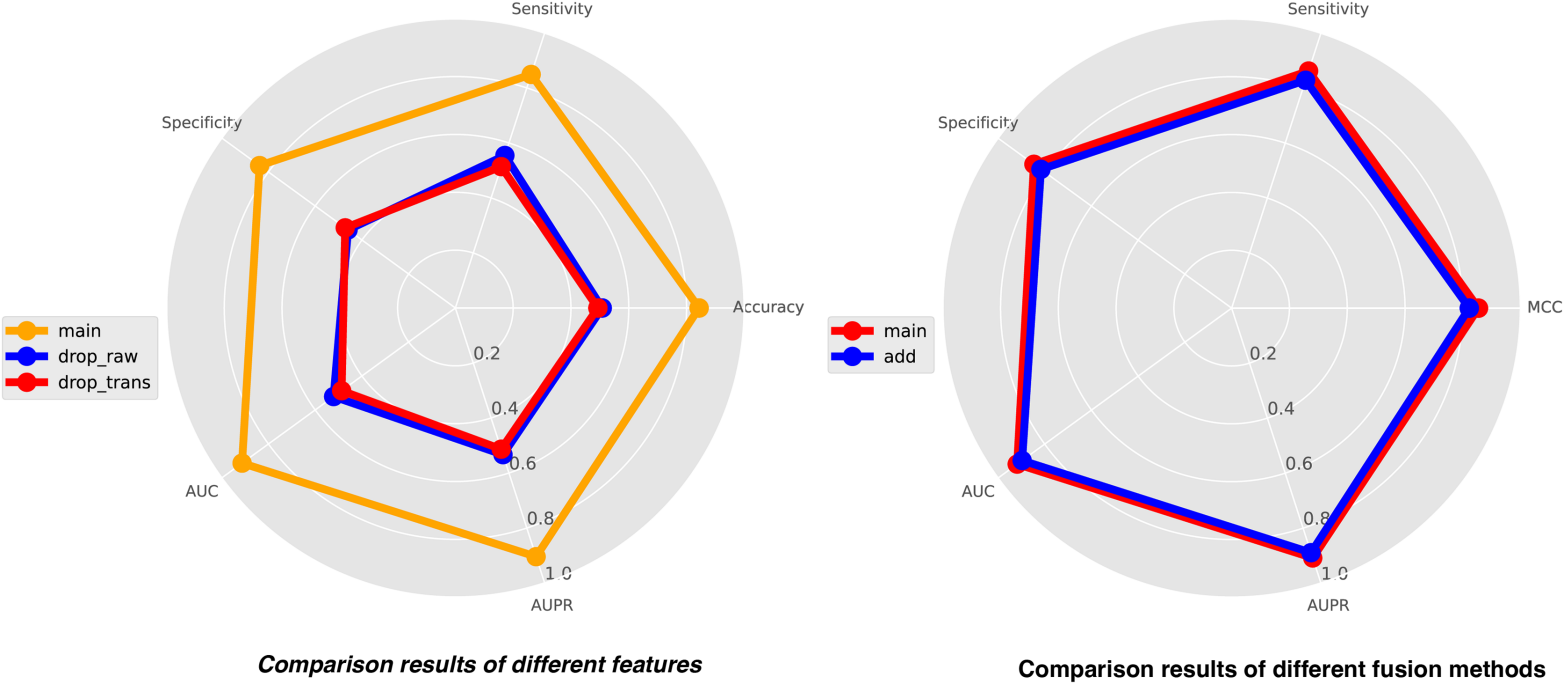
The comparison results of different features (left) and different fusion methods (right).

In this study, we integrate three types of features for comprehensive analysis, including feature representations from similarity matrices, feature representations from sequence structure diagrams, and k-mer sequence representations. K-mer representations are pre-coded representations based on the frequency count of sequence k-mer substrings, while the other two features are dynamically learned through neural networks. To achieve a holistic understanding of the sequences, we perform local-global integration of these features. To illustrate the importance of combining these three features, we construct two additional features, drop-raw and drop-trans, to validate our model’s performance. Drop-raw represents features without similarity matrix information, and drop-trans represents features without sequence structure graph information. The comparison results of the three types of features are presented in Fig. 5. Our model demonstrates the best recognition performance when all features are utilized. Drop-raw performs slightly better than drop-trans in terms of AUC and AUPR, suggesting that similarity matrices exert a stronger influence on the model compared to sequence structure graphs. Overall, all three types of features are essential and significantly enhance the effectiveness of site recognition. The local-global fusion block is a crucial component of our model, which integrates multiple features from both local and global perspectives by combining learned similarity matrix features with sequence-structure graph features. To demonstrate the necessity of this fusion block, we design another strategy using only weighted fusion, and the results are also presented in Fig. 5. The outcomes reveal that the recognition performance of the model is inferior when only weighted fusion is employed compared to the local-global fusion block. This underscores the significance of the local-global fusion block in achieving superior performance and highlighting its role in effectively capturing comprehensive sequence information.

### Performance comparison of predictors

In this study, we conduct a rigorous 10-fold cross validation to compare our model with five other existing methods on the A101 dataset. The compared methods include M6AMRFS, BERMP, RFAthM6A, BERT-m7G (Zhang et al., 2021), and the model designed by Le & Ho (2022). M6AMRFS encodes RNA sequences using two feature descriptors, dinucleotide binary coding, and local site-specific dinucleotide frequency. It enhances feature representation through the F-score algorithm combined with sequence forward search (SFS) and employs XGBoost as the downstream classifier. BERMP utilizes GRU to represent RNA sequences and adopts an end-to-end training process for site recognition. RFAthM6A attempts to classify various types of features derived from RNA sequences using machine learning methods. Our model, M6ATMR, adopts the transformer encoder to extract sequence representations and uses a stacking ensemble classifier for predicting m6A sites. We also consider two other transformer-based models, BERT-m7G, and the model designed by Le & Ho (2022). In their approaches, BERT-m7G uses bidirectional encoder representations from transformers (BERT) to extract sequence representations, while Le & Ho (2022) use a pre-trained transformer to explore features and a convolutional neural network for further feature extraction. The comparison results are presented in Table 3. Our model achieves an Acc value of 84.42% and a MCC value of 83.72%. These indicators demonstrate that our model outperforms the other five methods in most aspects. Compared to the other models, our approach exhibits a remarkable improvement, with a maximum of 11.09% higher accuracy and 16.13% higher MCC value. The model designed by Le & Ho (2022) has the lowest MCC value among all methods, while BERMP and RFAthM6A show similar performance across various indicators. However, we note that the specificity (Sp) value of our method is slightly lower than that of BERMP and RFAthM6A, indicating a minor deficiency in predicting true negative samples. Nevertheless, overall, the experimental results clearly demonstrate the effectiveness of our model, which stands out as a superior approach for m6A site prediction on RNA sequences.

**Table 3:** The comparison results of different recognition methods. N.A. denotes the value of the indicator is not provided by corresponding studies.

Models	Acc	MCC	Sn	Sp
M6ATMR	0.8434	0.6867	0.8488	0.83.77
M6AMRFS	0.8105	0.6210	0.8067	0.81.43
BERMP	N.A.	0.7260	0.8230	0.90.00
RFAthM6A	N.A.	0.7255	0.8222	0.90.00
BERT-m7G	0.8213	0.7023	0.8124	0.7985
Le & Ho (2022)	0.7325	0.5254	0.7234	0.6638

## Discussion and conclusion

In this article, we commence by reviewing classical methods for identifying RNA m6A modification sites and presenting our own perspectives. Subsequently, we analyze the limitations of these methods, leading us to propose a novel sequence-dependent-only RNA m6A modification site recognition method, named M6ATMR. M6ATMR utilizes the Transformer to guide the reconstruction of similarity matrices for each RNA sequence, thereby optimizing the feature representation of RNA sequences. Comparative analysis with other recognition methods reveals that M6ATMR demonstrates superior predictive performance, as evidenced by improved metrics. Comprehensive experiments further attest to the accuracy and robustness of our model. Additionally, we delve into several critical aspects. First, computing the similarity matrix and optimizing feature generation proves effective in enhancing the recognition performance of RNA m6A modification sites. Second, cooperative updating of similarity matrices and sequence structure graphs in the sequence representation of the same RNA sequence facilitates the retention of richer nucleotide distribution information. Third, the deep fusion of multiple features from both local and global perspectives results in a comprehensive understanding of RNA sequences.

However, there remain certain limitations in our study that warrant attention. First, the restriction of RNA sequence length necessitates the selection of the A101 dataset for model verification, rendering our approach less adept at handling short RNA sequences. Second, our current model primarily focuses on nucleotide distribution information, with limited exploration of other sequence properties. Future work will address these issues and explore the application of our model to the identification of other modification types, such as M1A, and modifications on DNA sequences.

## Supplemental Information

10.7717/peerj.15899/supp-1 Supplemental Information 1 Raw Data.

## Additional Information and Declarations

### Data Availability

The following information was supplied regarding data availability: Raw data are available in the Supplemental Files.
